# An integrative multi-omics framework for decoding microglial ecosystems in Alzheimer's disease

**DOI:** 10.1093/bib/bbag057

**Published:** 2026-03-10

**Authors:** Chuyun Zhang, Kei Hang Katie Chan

**Affiliations:** Department of BMS, City University of HK, HK SAR, China; Department of BMS, City University of HK, HK SAR, China; Department of EE, City University of HK, HK SAR, China; Dept of Epi & Biostat, Ctr for Global Cardiometabolic Hlth & Nutr, UCI, United States

## Abstract

**Background:**

Alzheimer’s disease involves complex cellular alterations, yet current methods analyze cell states, signaling, and genetic risk in isolation, preventing systems-level understanding.

**Methods:**

We developed an integrative framework combining quasi-binomial compositional analysis, scDemon [1], LIANA [2], scFates [3], and scDRS [4], applied to 12 integrated snRNA-seq datasets from human entorhinal and prefrontal cortex.

**Results:**

Analysis revealed coordinated cellular alterations with inhibitory neuron depletion and microglia expansion. scDemon identified novel microglial states including a filopedia dynamics module (MYO10/PARVG). Trajectory analysis showed progression from homeostatic (P2RY12-high) to proliferative (APOE, AXL-high) and senescent (CDKN1A-high) states. LIANA implicated RTN4-LINGO1 signaling in impaired neuronal repair, while scDRS mapped disease genetic risk to microglial cells.

**Conclusion:**

Our framework links cellular pathophysiology to genetic etiology, providing a blueprint for identifying therapeutic targets in neurodegenerative disease.

**References:**

1. Mathys H, Boix CA, Akay LA et al. ‘Single-cell multiregion dissection of Alzheimer’s disease.’ Nature 2024;632:858–868.

2. Dimitrov D, Schäfer PSL, Farr E et al. ‘LIANA+ provides an all-in-one framework for cell–cell communication inference.’ Nature Cell Biology 2024;26:1613–1622.

3. Faure L, Soldatov R, Kharchenko PV et al. ‘scFates: a scalable python package for advanced pseudotime and bifurcation analysis from single-cell data.’ Bioinformatics 2022;39.

4. Zhang MJ, Hou K, Dey KK et al. ‘Polygenic enrichment distinguishes disease associations of individual cells in single-cell RNA-seq data.’ Nature Genetics 2022;54:1572–1580.

